# Panacus: fast and exact pangenome growth and core size estimation

**DOI:** 10.1093/bioinformatics/btae720

**Published:** 2024-11-29

**Authors:** Luca Parmigiani, Erik Garrison, Jens Stoye, Tobias Marschall, Daniel Doerr

**Affiliations:** Faculty of Technology and Center for Biotechnology (CeBiTec), Bielefeld University, Bielefeld 33615, Germany; Department of Genetics, Genomics and Informatics, University of Tennessee Health Science Center, Memphis, TN 38163, United States; Faculty of Technology and Center for Biotechnology (CeBiTec), Bielefeld University, Bielefeld 33615, Germany; Institute for Medical Biometry and Bioinformatics, Medical Faculty and University Hospital Düsseldorf, Heinrich Heine University Düsseldorf, Düsseldorf 40225, Germany; Center for Digital Medicine, Heinrich Heine University Düsseldorf, Düsseldorf 40225, Germany; Center for Digital Medicine, Heinrich Heine University Düsseldorf, Düsseldorf 40225, Germany; Department for Endocrinology and Diabetology, Medical Faculty and University Hospital Düsseldorf, Heinrich Heine University Düsseldorf, Düsseldorf 40225, Germany; German Diabetes Center (DDZ), Leibniz Institute for Diabetes Research, Düsseldorf 40225, Germany

## Abstract

**Motivation:**

Using a single linear reference genome poses a limitation to exploring the full genomic diversity of a species. The release of a draft human pangenome underscores the increasing relevance of pangenomics to overcome these limitations. Pangenomes are commonly represented as graphs, which can represent billions of base pairs of sequence. Presently, there is a lack of scalable software able to perform key tasks on pangenomes, such as quantifying universally shared sequence across genomes (the *core genome*) and measuring the extent of genomic variability as a function of sample size (*pangenome growth*).

**Results:**

We introduce Panacus (pangenome-abacus), a tool designed to rapidly perform these tasks and visualize the results in interactive plots. Panacus can process GFA files, the accepted standard for pangenome graphs, and is able to analyze a human pangenome graph with 110 million nodes in <1 h.

**Availability and implementation:**

Panacus is implemented in Rust and is published as Open Source software under the MIT license. The source code and documentation are available at https://github.com/marschall-lab/panacus. Panacus can be installed via Bioconda at https://bioconda.github.io/recipes/panacus/README.html.

## 1 Introduction

The field of pangenomics emerged from the study of bacterial genomes (Tettelin *et al.* 2005), initially defining the pangenome as the complete set of genes within a species. This gene-based approach views the pangenome as the union of all genes across strains, distinguishing between the *core genome—*genes shared by all strains—and the *accessory genome—*genes found in one or more, but not all, strains.

Since the gene-based approach requires fully annotated genomes and excludes noncoding areas of the genome, an alternative definition of pangenome was proposed based purely on DNA sequences (Bentley 2009). In contrast to the gene-based view, a sequence-based approach represents a pangenome as the set of all nonredundant genomic sequences, including coding and noncoding regions, and seamlessly extends to eukaryotic genomes with their complex and larger genomes.

Regardless, both perspectives treat the pangenome as a set, either of genes or DNA sequences, allowing for the examination of genomic variability and similarities. Two concepts have been central to address these aspects: *pangenome growth* and the *core curve* (Tettelin *et al.* 2005). Pangenome growth measures the expansion of total genomic content as additional genomes are sequenced. This process starts with a single genome and incrementally includes new genomes, expanding the collective genomic repertoire, whether defined by sequences or genes. Since the order of genome inclusion can affect the growth curve, pangenome growth is defined as the average over all possible genome orders. Similarly, the core curve illustrates the average size of the core genome as additional genomes are added.

As pangenomics progressed, so did the representation of pangenomes, by retaining the order of genomic sequences through a graph (Marcus *et al.* 2014). This evolution led to the adoption of *sequence graphs*, where nodes represent sequences shared across genomes, and edges indicate the consecutive presence of these sequences within a genome (Paten *et al.* 2017). Sequence graphs have been extensively used for the genome assembly problem (Compeau *et al.* 2011), where they allow the reconstruction of the original sequence from large read sets.

On the other hand, sequence graphs are typically lossy, implying they can encode for a larger set of sequences than those they were initially constructed from, which makes them less attractive for a faithful representation of pangenomes. Augmenting a sequence graph with paths that correspond to the original sequences forms a *pangenome graph* [also referred to as variation graph (Garrison *et al.* 2018)], the primary target of our tool.

While plenty of tools are available to manipulate sequence graphs, e.g. gfatools (Li *et al.* 2020) or gfastats (Formenti *et al.* 2022), pangenome graphs are less represented. Moreover, with the increase in the amount of genomic data we need efficient tools to handle pangenome graphs.

Here, we introduce Panacus (pangenome-abacus), a tool designed for rapid extraction of information from pangenomes represented as pangenome graphs in the Graphical Fragment Assembly (GFA) format. Panacus not only efficiently generates pangenome growth and core curves but also provides estimates of the pangenome’s expansion with the addition of more genomes. In our study, Panacus is applied to generate the growth and core size for two pangenomes: HPRC-PGGB, a draft human pangenome graph from the Human Pangenome Reference Consortium (Liao *et al.* 2023), with a focus on euchromatic and exonic regions, and ECOLI-PGGB, a pangenome of 50 *Escherichia coli* genomes (Garrison *et al.* 2023).

## 2 Panacus

**Figure 1.**
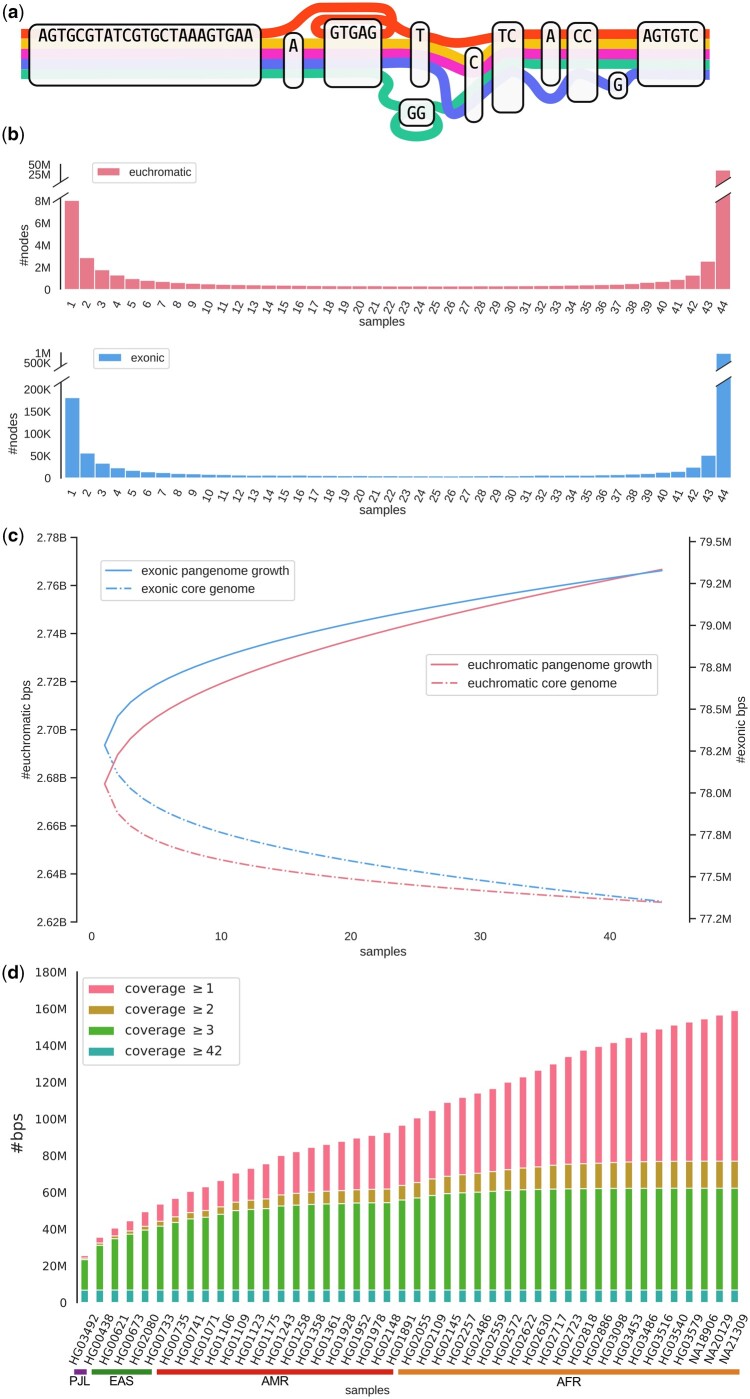
(a) A pangenome graph of five genomes. (b) Node coverage histograms of euchromatic and exonic base pairs in HPRC-PGGB. (c) Pangenome growth curves (solid) and core curves (dashed) of euchromatic and exonic base pairs in HPRC-PGGB, with the left axis scaling up to $2.90\times 10^{9}$ and the right axis to $83.5\times 10^{6}$. (d) Ordered growth histogram re-created from (Liao *et al.* 2023, Fig. 3g) of the nonreference (GRCh38) euchromatic genome in HPRC-PGGB of 44 samples representing four populations, Punjabi from Lahore, Pakistan (PJL), East Asian (EAS), Admixed American (AMR), and African (AFR).

Panacus is designed to count a variety of elements within pangenome graphs, including nodes, edges and base pairs that we will collectively refer to as *countables*. We define the *coverage* of a countable as the number of distinct paths that include that countable. For example, the coverage of the edge (TC, A) in Fig. 1a is four while the node GG has a coverage of one, despite the green path traversing the node more than once. Users can choose any type of countable to generate and visualize its coverage distributions.

A key feature of Panacus is its ability to quickly calculate pangenome growth and core curves from the coverage frequencies of countables. Furthermore, the tool allows to extract basic summary statistics or complete coverage tables for each countable. Finally, it produces an interactive report that contains the tabular data and its visualization within a standalone HTML page.

Panacus is designed to take as input a file in GFA format (https://github.com/GFA-spec/GFA-spec), where each line represents either a DNA segment (S-line), a link between two segments (L-line), or a path (P-line). In addition, we support walks (W-lines), which were introduced in a successive version (v1.1) of the GFA format. Paths, or walks, are internally represented as sets of countables, and their coverage is calculated based on the number of paths that contain at least one of those countables. Therefore, how many times a countable is encountered inside a path is not considered. This approach helps prevent the over representation of highly repeated countables, which could otherwise obscure the true growth (or core) of the graph. The only difference between P-lines and W-lines is in their parsing, the internal representation remains the same. In the subsequent text, we will not make any difference between P- and W-lines, but collectively refer to them as *paths*.

Since a path can represent multiple types of sequence, like a gene, a contig, or even an entire chromosome, Panacus offers the option to group paths together. Users can group paths by passing a file containing the mapping or they can be automatically grouped by samples or haplotype following the PanSN notation (https://github.com/pangenome/PanSN-spec). All features that are subsequently presented support grouped paths.

Another feature of Panacus is the possibility to focus on, or exclude, specific parts of the pangenome. It allows selecting regions meeting a minimum coverage threshold or matching path names or path regions in a BED file. To mediate between the coordinate systems of paths and their corresponding DNA sequences, Panacus recognizes coordinate subsetting also in path names of P-lines, e.g. path-id:10–200.

A possible approach to construct pangenome growth curves involves creating a set of countables for each path, sampling a random order of the paths, and sequentially including them. This process is repeated for several permutations of the order of paths, taking the average of the total number of new countables at each inclusion step and obtaining an approximate expected growth of the pangenome.

In contrast, Panacus adopts a method similar to the one presented in Parmigiani *et al.* (2024) to efficiently calculate the pangenome growth and the core curve. This approach is both *exact* and efficient. It avoids sampling and repetitive computation of the union of paths, thereby saving both memory and time. Notably, this concept is not new and has been recognized in ecology since the 1970s, but it remains largely underutilized in bioinformatics. Parmigiani *et al.* (2024) applied this concept to show how *k*-mers (sequences of length *k*) found in multiple genomes yield a pangenome growth similar to that of genes. Here, Panacus uses this method to efficiently generate pangenome growth and core curves for countables within the paths of pangenome graphs.

The principle behind this approach is as follows: pangenome growth can be equivalently defined as either the average size of the incremental union of paths across all possible orders or the average size of the union of all combinations of *m* paths, where *m* goes from 1 to the total number of paths. According to the second definition, a countable contributes to the average each time the union of paths includes *at least* one of the paths that contains the countable. This means that the greater the coverage of a countable—the more paths are passing through it—the more it contributes to the average, since it is present in more combinations of *m* paths. The contribution of a countable can be calculated by subtracting from the number of combinations of *m* paths how many *do not* contain the countable. For example, if *m *=* *3 and there are 10 paths with only 4 containing the countable, there are $\left(\begin{array}{c} 10\\ 3 \end{array}\right)-\left(\begin{array}{c} 10-4\\ 3 \end{array}\right)$ combinations of paths where at least one of these four paths is present.

The same strategy applies to calculating the core curve, counting the contribution of a countable only if it is present in all *m* paths. In addition, the definition of the core genome can be relaxed to encompass countables that are present in a certain percentage of paths. This percentage is determined by the user-defined *quorum* parameter, *q*, which can range from 0 to 1, specifying the proportion of paths in which a countable must be present to be considered part of the core.

A key data structure of the algorithm is the histogram *h*(*i*), as depicted in Fig. 1b, which tracks the *coverage frequency* of the pangenome graph. This histogram stores the number of countables that appear in exactly *i* paths and it can be used to directly calculate the expected growth and core curves for each subset size *m*.

While accurately extrapolating pangenome growth and core genome size for new, unseen genomes is challenging, Panacus aids in making an educated estimate. To facilitate this, we provide a Python script within Panacus that enables users to extrapolate the growth and core curves using both parametric methods, such as power law (Tettelin *et al.* 2008) or exponential decay (Tettelin *et al.* 2005) and a nonparametric method, the Chao2 estimator (Chao 1987).

## 3 Results

We study the pangenome growth of the draft human pangenome graph hprc-v1.0.pggb (HPRC-PGGB) recently released by the *Human Pangenome Reference Consortium* (Liao *et al.* 2023), and graph ecoli50 (ECOLI-PGGB) that was published in Garrison *et al.* (2023). Both graphs have been constructed with *PanGenome Graph Builder* (pggb) (Garrison *et al.* 2023). The former graph constitutes the complete haplotype-resolved assemblies of 44 samples and reference assemblies *GRCh38* and *T2T-CHM13v1.1*, and encompasses overall 8.41 gigabases distributed over 111 million nodes. The latter graph is composed of genome assemblies from 50 *Escherichia coli* strains and consists of over 18 megabases and 1.5 million nodes.

We benchmark Panacus against a competing tool called odgi heaps (Guarracino *et al.* 2022) that is able to process pangenome graphs stored in GFA v1.0 format. This tool’s method is based on sampling permutations and as such produces only an estimate of the growth curve. Obtaining stable estimates of pangenome growth with odgi heaps depends on the number of generated samples. This affects the running time, which is further prolonged by the preceding construction of an index of the GFA file, unless already present. For these reasons, it is difficult to devise a fair comparison between the two methods. Table 1 reports the running time and memory consumption for both methods on the two graphs. We ran odgi heaps twice, once sampling only one permutation and another time sampling 100 permutations. While a single sample is generally insufficient to give a stable estimate, an average over 100 samples results in an acceptable estimation of the pangenome growth curve for the dataset at hand.

**Table 1.** Comparison of CPU time (h:mm:ss) and maximum resident set size (GB) between Panacus and odgi heaps in computing pangenome growth curves on base pairs for HPRC-PGGB and ECOLI-PGGB.

Dataset	Tool	Time	Memory	Method
HPRC-PGGB	odgi build	5:01:56	182.41	Indexing
	odgi heaps	0:23:46	151.72	1 perm.
		47:13:54	148.89	100 perms.
	Panacus	0:54:40	51.03	Exact
ECOLI-PGGB	odgi build	0:49	1.53	Indexing
	odgi heaps	0:07	0.64	1 perm.
		11:06	0.64	100 perms.
	Panacus	0:13	0.36	Exact

For HPRC-PGGB, the computation time for the pangenome growth step with odgi heaps is approximately 24 min, notably less than half the time required by Panacus, which takes nearly 55 min. However, when including the time for odgi’s graph indexing (around 5 h), the total computation time for odgi heaps increases significantly, making it about five times slower than Panacus. In addition, when odgi heaps processes 100 permutations, its completion time extends dramatically to nearly 47 h, becoming a major time-consuming step. In terms of memory usage, odgi requires between 148 and 182 GB, whereas Panacus is much more memory-efficient, needing only 51 GB, which is approximately a third of odgi’s requirement. These trends are consistent with the benchmarks on the ECOLI-PGGB graph, where the overall running times for odgi with 100 permutations, indexing included, accumulate to 11:55 min and for Panacus to 13 s.

Panacus also provides additional functionality not offered by odgi heaps, such as quantifying countables other than base pairs, and the simultaneous calculation of multiple other statistics, including core curves. A comparison of the features between odgi heaps and Panacus is shown in Table 2.

**Table 2.** Comparison between methods.

	Parmigiani *et al.* (2024)	odgi heaps	Panacus
Input format	FASTA	GFA v1.0/OG	GFA v1.1
Input type	Sequences	Pangenome graph	Pangenome graph
Countables	*k*-mers	bps	Nodes/edges/bps
Growth calculation	Exact	Sampling	Exact
Core curve calc.	Exact	✗	Exact
Quorum core curve calc.	Exact	✗	Exact
Ordered growth calc.	✗	✗	*✓*
Path grouping	✗	*✓*	*✓*
Coverage thresholding	✗	*✓*	*✓*
Subsetting	✗	*✓*	*✓*
Exclusion	✗	✗	*✓*

Panacus’ capabilities of subsetting pangenome graphs enables direct analysis of specific region sets, rendering tedious manipulation of the graph’s GFA file unnecessary. We demonstrate this functionality by comparing the pangenome growth of euchromatic and exonic regions of autosomal chromosomes of the human pangenome. By restricting to euchromatic sequence, we seek to avoid any error in our analysis caused by under-alignment. Here we use coordinates of euchromatic and exonic regions provided by Liao *et al.* (2023) to calculate node coverage histograms (Fig.1b), and base pair-based pangenome growth and genome core curves (Fig. 1c). To extract exonic regions, we selected exon annotations whose transcript biotype was set to protein coding. Both, euchromatic and exonic sequence, exhibit a convex coverage pattern and similar pangenome growth and core curves. Yet, the euchromatic sequence has a slightly higher ratio of shared nodes compared to unique nodes (0.22 versus 0.18), and also the relative coverage in intermediate ranges (2–43 samples) is higher in euchromatic sequence compared to exonic by 32%, showing a relatively higher degree of variability in the genomic sequences of the human pangenome at hand. This affects the euchromatic pangenome growth curve to have a marginally higher slope, indicating a slightly elevated growth compared to the exonic pangenome. Further plots included the Appendix S1 show coverage histogram and pangenome growth for all chromosomes including X and Y, as well as for the sex chromosomes alone.

At last, Panacus can also produce ordered growth histograms and was used to illustrate the growth of nonreference, euchromatic autosomal sequences of the human pangenome in (Liao *et al.* 2023, Fig. 3g), a re-creation of which is shown in Fig. 1d.

## 4 Conclusion

We have introduced Panacus, a versatile and efficient tool designed for exploring and comparing pangenome graphs. Panacus enables the rapid generation of pangenome growth and core curves directly from GFA files. In this paper, we demonstrated its use in comparing distinct areas within a human pangenome graph. Beyond this application, Panacus can also be used to compare the same pangenome constructed using different tools, or to analyze completely different pangenomes, assessing their growth and core sizes.

## Supplementary Material

btae720_Supplementary_Data

## References

[btae720-B1] Bentley S. Sequencing the species pan-genome. Nat Rev Microbiol 2009;7:258–9. 19287447 10.1038/nrmicro2123

[btae720-B2] Chao A. Estimating the population size for capture-recapture data with unequal catchability. Biometrics 1987;43:783–91. 3427163

[btae720-B3] Compeau PEC , Pevzner PA, Tesler G et al How to apply de Bruijn graphs to genome assembly. Nat Biotechnol 2011;29:987–91. 22068540 10.1038/nbt.2023 PMC5531759

[btae720-B4] Formenti G , Abueg L, Brajuka A et al Gfastats: conversion, evaluation and manipulation of genome sequences using assembly graphs. Bioinformatics 2022;38:4214–6. 35799367 10.1093/bioinformatics/btac460 PMC9438950

[btae720-B5] Garrison E , Sirén J, Novak AM et al Variation graph toolkit improves read mapping by representing genetic variation in the reference. Nat Biotechnol 2018;36:875–9. 30125266 10.1038/nbt.4227 PMC6126949

[btae720-B6] Garrison E, Guarracino A, Heumos S et al Building pangenome graphs. bioRxiv. 2023.

[btae720-B7] Guarracino A , Heumos S, Nahnsen S et al ODGI: understanding pangenome graphs. Bioinformatics 2022;38:3319–26. 35552372 10.1093/bioinformatics/btac308 PMC9237687

[btae720-B8] Li H , Feng X, Chu C et al The design and construction of reference pangenome graphs with minigraph. Genome Biol 2020;21:265. 33066802 10.1186/s13059-020-02168-z PMC7568353

[btae720-B9] Liao W-W , Asri M, Ebler J et al A draft human pangenome reference. Nature 2023;617:312–24. 37165242 10.1038/s41586-023-05896-x PMC10172123

[btae720-B10] Marcus S , Lee H, Schatz MC et al SplitMEM: a graphical algorithm for pan-genome analysis with suffix skips. Bioinformatics 2014;30:3476–83. 25398610 10.1093/bioinformatics/btu756 PMC4253837

[btae720-B11] Parmigiani L , Wittler R, Stoye J. Revisiting pangenome openness with *k*-mers. PCI Commun J 2024;4:e47.

[btae720-B12] Paten B , Novak AM, Eizenga JM et al Genome graphs and the evolution of genome inference. Genome Res 2017;27:665–76. 28360232 10.1101/gr.214155.116 PMC5411762

[btae720-B13] Tettelin H , Masignani V, Cieslewicz MJ et al Genome analysis of multiple pathogenic isolates of *Streptococcus agalactiae*: implications for the microbial “pan-genome. Proc Natl Acad Sci USA 2005;102:13950–5. 16172379 10.1073/pnas.0506758102 PMC1216834

[btae720-B14] Tettelin H , Riley D, Cattuto C et al Comparative genomics: the bacterial pan-genome. Curr Opin Microbiol 2008;11:472–7. 19086349 10.1016/j.mib.2008.09.006

