# Supplementary material for: Panacus: fast and exact pangenome growth and core size estimation
Source: Bioinformatics. 2024 Nov 29;40(12):btae720. doi: 10.1093/bioinformatics/btae720 (PMC11665632; doi:10.1093/bioinformatics/btae720)
Supplement: btae720_Supplementary_Data [file btae720_supplementary_data.pdf]

Panacus: fast and exact pangenome growth and core size estimation
Appendix

Luca Parmigiani, Erik Garrison, Jens Stoye, Tobias Marschall and Daniel Doerr

Coverage histograms of HPRC-PGGB graph

Complete genome (autosomes and sex chromosomes)

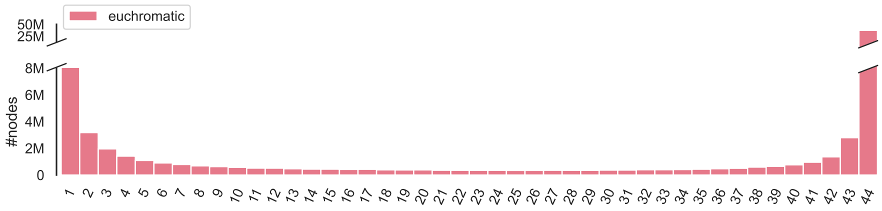

Figure 1: Node coverage histogram of autosomes and sex chromosomes in *euchromatic* regions.

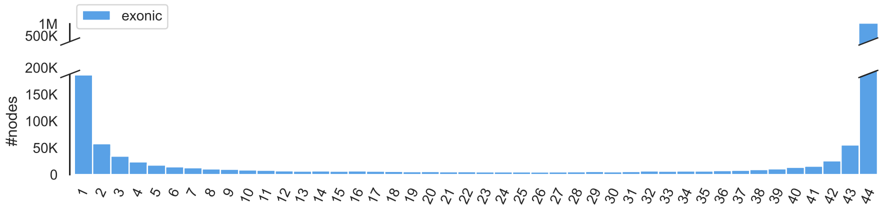

Figure 2: Node coverage histogram of autosomes and sex chromosome in *exonic* regions.

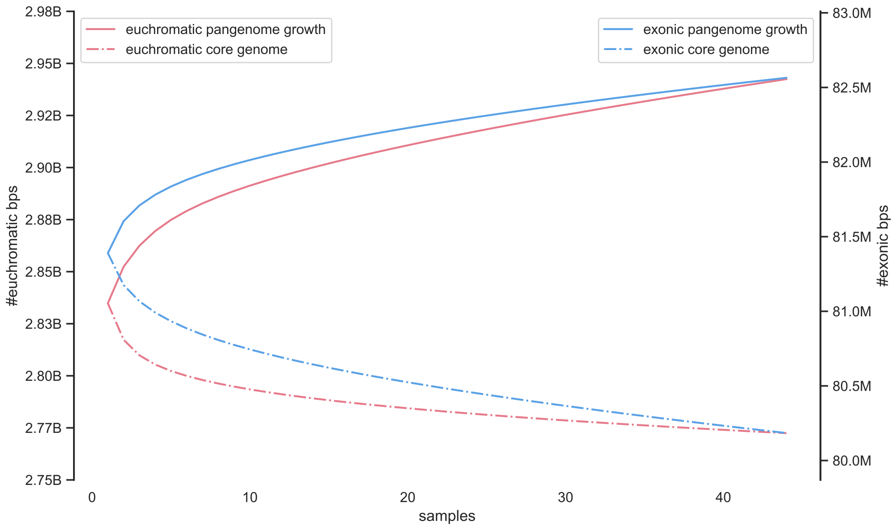

Figure 3: Euchromatic and exonic pangenome growth and core curve in autosomes and sex chromosomes.

Sex chromosomes

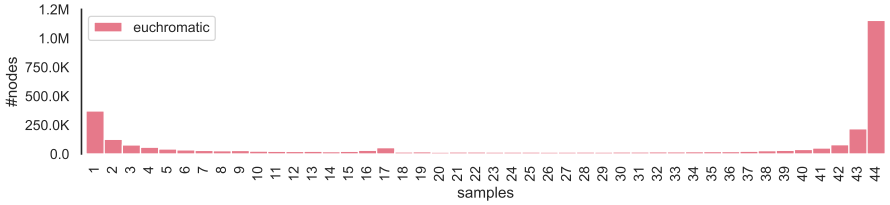

Figure 4: Node coverage histogram of sex chromosomes in *euchromatin* regions.

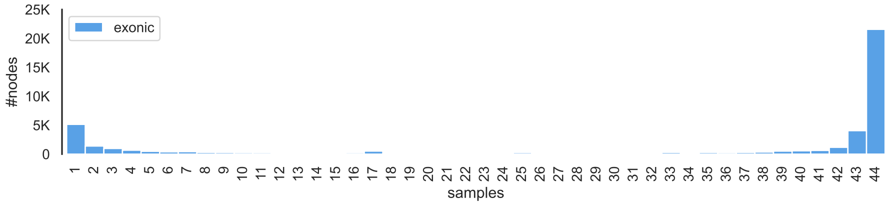

Figure 5: Node coverage histogram of sex chromosomes in *exonic* regions.

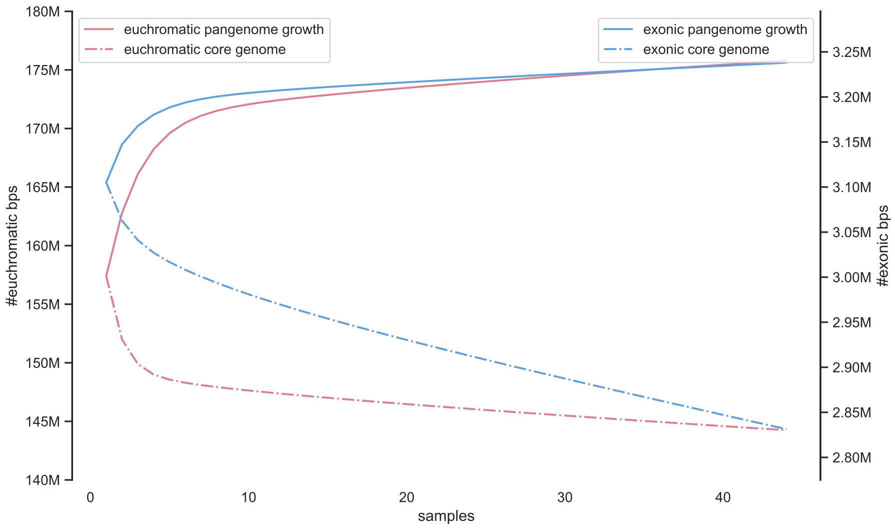

Figure 6: Euchromatic and exonic pangenome growth and core curve in sex chromosomes.
